# Retraction: Body lice of homeless people reveal the presence of several emerging bacterial pathogens in northern Algeria

**DOI:** 10.1371/journal.pntd.0014290

**Published:** 2026-05-05

**Authors:** The PLOS Neglected Tropical Diseases Editors

The *PLOS Neglected Tropical Diseases* Editors retract this article [1,2] due to concerns about compliance with the PLOS Human Subjects Research policy.

The Materials and methods subsection Ethics statement and louse sampling in [1] reports that the study involved body lice specimen collected between September 2014 and June 2016 from the clothes of homeless people in Algeria, and that the study was approved by the Centre d’Accureil pour Personnes sans Domicile Fixe, Algeria, and the Social Service d’Aide Médicale Urgente, Algeria. The article does not report ethics approval document reference numbers.

Co-author PP stated that there are no ethics approval reference numbers to report as the approvals were provided in the form of a stamp and signature on the ethics approval request letter. They provided three copies of such approval requests for editorial review.

A representative of the Aix-Marseille Université Ethics Committee stated that the institutional investigation into the ethics concerns concluded this article meets ethical standards. They commented that the study described in [1] did not involve invasive sampling as the study is based solely on lice collected from the clothes of homeless people. The institute provided the same documentation for editorial review.

PLOS reviewed the documentation provided by co-author PP and the institute, and concluded that the documents did not fully resolve the concerns. Specifically,

- The documents provided for editorial review do not appear to be protocols submitted for ethics review or documents providing ethics approval, but instead they appear to be requests for permission from the director of the emergency shelters to carry out a study involving the collection of lice from the clothes of homeless people housed in the center.
- Although permission to carry out the study at the center was granted by the director of the emergency shelter, PLOS remains concerned about the lack of evidence of formal ethics approval for a study involving a vulnerable, homeless, and mostly illiterate population.
- The earliest documents provided for editorial review are dated October 1, 2014, and March 3, 2015, whereas the article reports that sample collection started in September 2014, suggesting that the approval was not granted prospectively. The *PLOS Neglected Tropical Diseases* policy for Human Subjects Research states that studies involving human participants must obtain prior ethics approval.

ML, IB, PP, FF, and OM did not agree with the retraction. NM, MD, and DR either did not respond directly or could not be reached.
